# Surgical decision–making in borderline resectable pancreatic cancer in the era of neoadjuvant therapy: from response assessment to individualized operative strategy

**DOI:** 10.3389/fsurg.2026.1935508

**Published:** 2026-09-08

**Authors:** Lei Zhu, Hongbin Han, Gang Liu, Haichun Zhao, Mengxin Luo

**Affiliations:** 1 Department of General Surgery, Panjin Liao-Oil Field Gem Flower Hospital, PanJin, LiaoNing, China; 2 Department of Oncological Surgery, Panjin Liao-Oil Field Gem Flower Hospital, PanJin, LiaoNing, China; 3 Department of Quality Management, Panjin Liao-Oil Field Gem Flower Hospital, PanJin, LiaoNing, China

**Keywords:** borderline resectable, conversion surgery, neoadjuvant therapy, pancreatic cancer, pancreatic ductal adenocarcinoma

## Abstract

**Background:**

Neoadjuvant therapy (NAT) has become central to the management of borderline resectable pancreatic ductal adenocarcinoma (BRPC). This surgical narrative review offers a contemporary synthesis of evidence and guidelines, with emphasis on pretreatment staging, post-NAT surgical selection, intraoperative vascular tactics, and postoperative care.

**Methods:**

We conducted a structured narrative review of randomized and prospective studies, high-quality observational cohorts, and major international guidelines published through 31 December 2025. A systematic search of PubMed was performed, and key data were synthesized to inform a practical decision-making framework.

**Results:**

Although BRPC remains defined largely by vascular anatomy, biological and conditional factors are increasingly incorporated into decision-making. NAT is preferred initially, with resection reserved for carefully selected responders. For post-NAT restaging, contrast-enhanced CT and CA19-9 kinetics are the mainstays, while FDG-PET, DWI-MRI, radiomics, and biomarkers serve as problem-solving adjuncts when findings are equivocal. Surgical exploration is guided by physiologic recovery, absence of metastatic progression, and multidisciplinary consensus. Staging laparoscopy remains useful for detecting occult metastases. Intraoperative vascular resection is margin-driven, not routine: venous resection is standard, arterial resection is selective, and periarterial divestment spares the artery in some cases. Short-term surgical morbidity does not appear to be increased by NAT, yet the absence of uniform protocols for post-reconstruction anticoagulation represents an important area for future investigation.

**Conclusions:**

NAT has fundamentally transformed the management of BRPC, establishing a surgical pathway that is dictated by tumor biology and sequentially timed according to treatment response and clinical course. Optimizing outcomes ultimately depends on standardized restaging protocols, judicious selection of patients who truly benefit from resection, and the concentration of complex vascular procedures in high-volume, experienced centers. A practical algorithm is proposed to synthesize these decision points for clinical use.

## 1 Introduction

Pancreatic cancer (PC) ranks third among cancer-related deaths, with a 5-year relative survival below 10%—and under 8% for pancreatic ductal adenocarcinoma (PDAC), which accounts for 90% of cases (1, 2). Curative-intent resection followed by adjuvant therapy is feasible in only 15%–20% of patients; nearly half present with metastases, and a substantial proportion have borderline resectable disease (BRPC) (1, 3).

In BRPC, primary surgery often fails due to technical obstacles to R0 clearance with vascular encasement and a high rate of occult micrometastases (4, 5). Neoadjuvant therapy (NAT) has therefore become integral to BRPC management (6). As NAT enters routine practice, the central challenge has shifted from whether to treat preoperatively to how to make consistent surgical decisions after treatment. Restaging after NAT often yields discordant signals among imaging, biomarkers, and clinical recovery, complicating response assessment. Operative planning must incorporate tailored vascular strategies—venous resection/reconstruction being increasingly routine, while arterial involvement demands highly selective approaches. Perioperative care must balance thrombotic and bleeding risks, particularly after venous reconstruction, where anticoagulation remains non-standardized. Current guidelines provide broad principles but allow substantial center-to-center variability.

From a surgical standpoint, this narrative review consolidates current evidence and clinical recommendations regarding NAT for BRPC, and distills them into a practical framework addressing pretreatment stratification, post-NAT restaging, intraoperative vascular management, and perioperative risk control.

## 2 Methods

We conducted a narrative review with a structured search of PubMed from inception through 31 December 2025, for peer-reviewed, English-language human studies. The search combined MeSH terms and free-text keywords: [“pancreatic neoplasms” (MeSH) OR “pancreatic ductal adenocarcinoma” OR “pancreatic cancer”] AND [“neoadjuvant therapy” (MeSH) OR “preoperative therapy” OR “neoadjuvant treatment”] AND (“borderline resectable” OR “locally advanced”) AND [“surgical procedures, operative” (MeSH) OR “pancreatectomy” OR “vascular resection” OR “venous resection” OR “arterial resection”]. We also screened reference lists of included articles and major guidelines.

We included randomized controlled trials, prospective/retrospective cohorts, registry analyses, meta-analyses, and society guidelines/consensus documents informing NAT decisions for BRPC. We excluded case reports, editorials, letters, conference abstracts, non-English studies, and those focusing on non-PDAC tumors. Additional exclusion criteria comprised: (1) studies with fewer than 10 patients; (2) studies lacking clearly defined BRPC criteria or using non-standardized definitions; (3) studies without extractable outcome data for NAT-treated BRPC patients; (4) studies focusing exclusively on biomarkers or imaging without clinical outcome correlation; and (5) duplicate publications from the same cohort without additional follow-up.

Evidence synthesis was organized around key surgical decision themes, considering study design, sample size, and relative quality. Given the narrative nature of this review, formal quality assessment (e.g., QUADAS-2, ROBINS-I) and meta-analytic pooling were not performed; readers should consider these limitations when applying the evidence clinically.

## 3 Pretreatment classification

The classification of BRPC is fundamentally defined by the anatomic relationship between the primary tumor and the adjacent major vasculature—including the superior mesenteric artery (SMA), celiac axis (CA), common hepatic artery (CHA), portal vein (PV), and superior mesenteric vein (SMV). Although specific vessel involvement criteria vary across consensus guidelines (7), the NCCN definitions remain the most widely adopted framework in surgical practice (8). Recognizing that anatomy alone does not fully capture clinical complexity, the MD Anderson group was the first to expand this paradigm by incorporating biological factors (most notably elevated CA19-9) and conditional factors such as performance status and comorbidities (9). This broader perspective acknowledged that even patients with anatomically resectable disease might benefit more from NAT due to occult systemic progression risk or limited physiological reserve. Building on this concept, the IAP formally codified a three-domain classification in 2017: (1) anatomical vessel involvement, (2) biological risk (CA19-9 > 500 U/mL or biopsy/PET-CT-confirmed regional nodal metastasis), and (3) patient-related conditional factors (ECOG PS ≥2) (10). This multidimensional framework has since been adopted by major organizations, including ESMO (11). The NCCN guidelines, however, have not formally incorporated biological or conditional domains into their BRPC definition, though they continue to emphasize performance status as a key determinant in treatment selection.

## 4 NAT in BRPC

Current guidelines (NCCN, ASCO, ESMO, CSCO, JPS) uniformly recommend NAT as the preferred initial approach for BRPC, with FOLFIRINOX (or mFOLFIRINOX) and gemcitabine-nab-paclitaxel (GnP) as preferred regimens, though no single standard is endorsed (8, 11–15).

### 4.1 Evidence, guidelines, and dynamic selection

Randomized trials since 2018 have increasingly favored NAT over upfront surgery in BRPC (16–19). The ESPAC-5 trial showed 8-week NAT markedly improved 1-year OS over immediate resection (39% upfront vs. 78%–84% for NAT regimens) (19). The PREOPANC trial demonstrated gemcitabine-based NAT achieved higher 5-year OS (20.5% vs. 6.5%) and improved intention-to-treat (ITT) R0 resection rates (41% vs. 28%) (18). A 2025 meta-analysis confirmed an OS benefit for BRPC (HR = 0.60, 95% CI, 0.38–0.96), but not for clearly resectable tumors (20). The PC-CURE-1 cohort reported median OS of 34.4 months for resected patients after NAT vs. 19.8 months for non-resected patients (21). Collectively, these findings support conversion surgery for rigorously selected responders after MDT evaluation.

### 4.2 Regimen-specific considerations and impact on surgical outcomes

Beyond the choice between NAT and upfront surgery, the selection of specific regimens also critically affects outcomes. Comparative data on regimen selection in BRPC remain limited. A 2022 systematic review and meta-analysis of BRPC/LAPC patients found that FOLFIRINOX was associated with higher resection and R0 resection rates, as well as better OS, compared with GnP (22). However, the NUPAT-01 randomized phase II trial (*n* = 51) reported an R0 resection rate of 67.4% with no significant difference between FOLFIRINOX and GEM/nab-PTX (23). A real-world single-center analysis further suggested that FOLFIRINOX offered a survival benefit in BRPC but with higher toxicity (24).

Regimen choice also influences surgical outcomes. Regimens incorporating chemoradiotherapy induce more pronounced fibrosis than chemotherapy alone (73% vs. 38%, *P* = 0.0001), which may complicate dissection but correlates with negative margins (25). The protective effect against postoperative pancreatic fistula (CR-POPF) is strongest with radiation-containing regimens, while the PREOPANC-2 trial reported similar major complication rates between FOLFIRINOX and chemoradiotherapy (26). Optimal timing of surgery may be regimen-dependent, with radiotherapy-containing protocols generally favoring earlier intervention. Within this framework, NAT functions both as therapeutic intervention and dynamic selection tool, identifying patients most likely to benefit from resection while sparing others from unnecessary morbidity.

## 5 Assessing response after NAT: a multimodal decision framework

Evaluating tumor response following NAT plays a pivotal role in identifying patients who are most likely to benefit from resection while sparing others from futile exploration. The initial and most critical determinant is the patient's overall clinical recovery—encompassing performance status, abatement of treatment-related adverse effects, and nutritional adequacy (with special attention to sarcopenia)—all of which bear well-established prognostic implications and must fundamentally condition the decision to proceed with surgery (27–29). From an oncological standpoint, therapeutic decisions are guided by a comprehensive synthesis of cross-sectional imaging findings and biological markers, enabling a practical and balanced assessment of whether to proceed with or forego exploration.

### 5.1 Post-NAT CT restaging: limitations and value of tumor shrinkage

In current guidelines and routine care, contrast-enhanced CT remains the primary tool for reassessment after NAT. Nevertheless, its morphological evaluation has restricted accuracy in predicting R0 resectability and surgical margins, a limitation attributable mainly to treatment-related fibrosis and vascular alterations (30, 31). The inherent imprecision is underscored by a 2021 meta-analysis, wherein a strict resectable-only criterion (*n* = 217) yielded a sensitivity of only 45% for R0 prediction, albeit with a specificity of 85%; upon inclusion of borderline resectable cases (*n* = 160), sensitivity improved to 81%, but specificity concomitantly dropped to 42% (32). Taken together, these data underscore the importance of interpreting CT findings within the broader clinical and biological context, and advise against relying exclusively on a lack of radiographic tumor regression to preclude surgical exploration.

### 5.2 Advanced CT techniques: from radiomics to functional imaging

Multiple CT-based tools—including radiomics, delta radiomics, dual-energy CT (DECT) iodine mapping, and perfusion imaging—consistently outperform conventional morphologic evaluation for post-NAT restaging, with radiomics models achieving AUCs of 0.89–0.94 for predicting pathological response and margin status, and DECT-derived extracellular volume fraction showing AUCs around 0.80 for discriminating responders (33–37). Encouragingly, externally validated delta-radiomics models incorporating clinical variables have improved prediction of margin status and survival over clinical-only models (35), and other radiomics approaches have shown promise in predicting pathological response and distant metastasis (34, 38). However, lack of standardized acquisition and segmentation protocols, alongside predominantly single-center data, has limited clinical acceptance (38). At present, these advanced imaging biomarkers should be viewed as adjunctive to, rather than determinative of, treatment decisions; broader adoption will require protocol harmonization and prospective multicenter validation.

### 5.3 Diffusion-weighted MRI: uncertainty and divergent evidence

The feasibility of diffusion-weighted imaging (DWI) for assessing treatment response after NAT remains inconclusive. A systematic review and meta-analysis (seven studies, *n* = 161) found that apparent diffusion coefficient (ADC) values showed extensive overlap between responders and non-responders, precluding definitive clinical recommendations (39). More recent evidence remains sharply divided: one study of 72 BRPC patients reported excellent discriminatory performance (AUC = 0.94) for a multiparametric DWI model incorporating ADC changes (40), whereas a larger retrospective cohort (*n* = 103) found no significant association between ADC changes and R0 margin status or histopathological regression grade (41). This discordance likely reflects differences in study design, patient selection, ADC measurement techniques, or threshold definitions—all warranting systematic investigation. At present, DWI should be considered an investigational adjunct rather than a standard component of post-NAT restaging.

### 5.4 FDG-PET: beyond morphology, toward functional biomarkers

FDG-PET-derived metabolic response provides a robust surrogate of viable tumor burden and surpasses conventional imaging for predicting long-term outcomes. In a large BRPC cohort, post-NAT metabolic response was the most potent preoperative predictor of major pathological response, outperforming CA19-9 (AUC 0.86 vs. 0.75), and retained independent prognostic value for survival (42). Integrating PET with MRI enhances resectability prediction compared with CT alone (43). Among PET metrics, total lesion glycolysis (TLG) is promising by integrating metabolic intensity with tumor burden. FDG-PET serves as a biologically anchored adjunct when CT or biomarker findings are equivocal. However, routine incorporation as a standard gatekeeper for surgical exploration requires standardized thresholds, consistent reporting, and prospective validation (42, 43).

### 5.5 Post-NAT response assessment: biomarkers, dynamics, and decision framework

CA19-9 is the most thoroughly investigated serum biomarker for post-NAT response assessment, with NCCN and ESMO guidelines advocating interpretation of its dynamics—sustained decline or normalization—alongside imaging and clinical status (8, 11). A 2025 meta-analysis (92 studies) identified post-NAT CA19-9 normalization as among the strongest independent predictors of OS, comparable to R0 resection and node status (44). Optimal thresholds vary by population: Japanese cohorts validate a post-NAT cut-off of <100 U/mL (45), while a 2024 multicenter analysis (*n* = 492) identified 202 U/mL (pre-NAT) and 78 U/mL (post-NAT) as empirically optimized thresholds (46); a 2025 cohort defined “favorable responders” as normalization or post-NAT <296 U/mL plus >40% reduction, associated with significantly better survival (47). Persistently elevated post-NAT CA19-9 signals unfavorable biology and shorter OS, with postoperative rises indicating early recurrence (48). For the 5%–10% of CA19-9 non-secretors, CA125 and CEA serve as alternatives (8, 49). ctDNA has shown promise in detecting minimal residual disease, with mutant KRAS elimination correlating with extended OS, though evidence remains limited (50–52); elevated CEA also predicted failure to reach surgery in a BRPC cohort (53). These biomarkers remain investigational, requiring validation in large, standardized cohorts.

**Figure 1:**
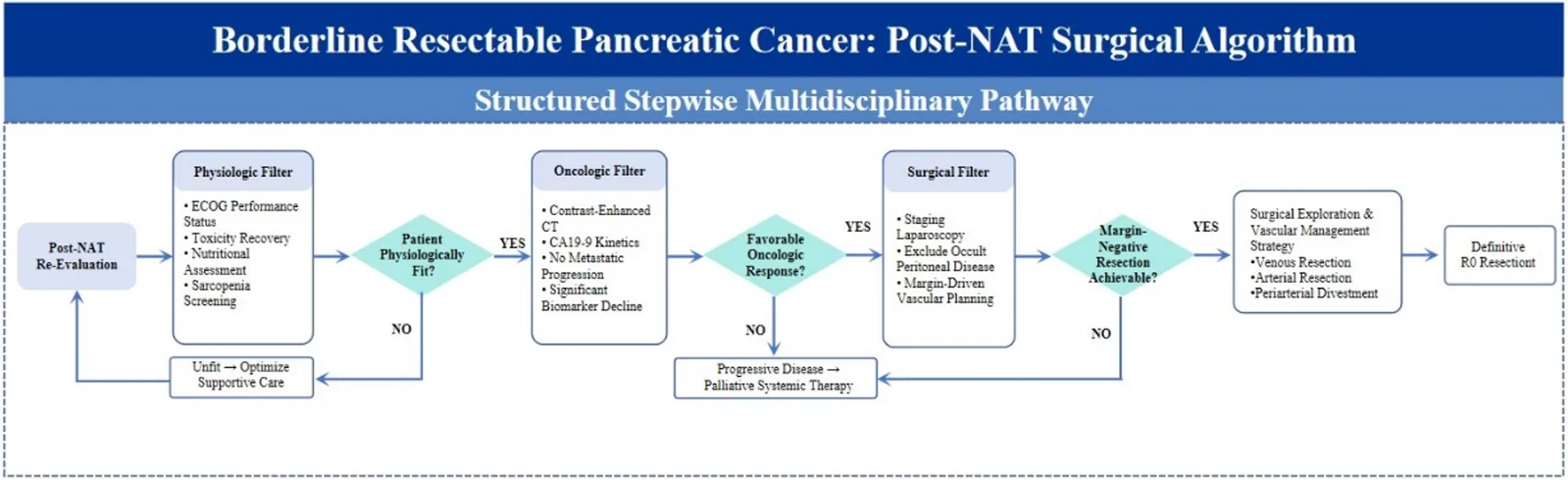
Post-NAT decision algorithm for surgical exploration in BRPC. The algorithm proceeds through three sequential filters: physiologic recovery (performance status, toxicity resolution, nutritional adequacy), oncologic reassessment (contrast-enhanced CT and CA19-9 kinetics as mainstays; FDG-PET and DWI-MRI as adjuncts when findings are equivocal), and staging laparoscopy to exclude occult peritoneal disease. Surgical exploration proceeds with a margin-driven vascular strategy. This framework is intended as a practical guide for multidisciplinary decision-making and should be adapted to individual patient circumstances and institutional expertise.

Based on this evidence, we propose a stepwise algorithm for post-NAT surgical exploration (Figure 1). First, verify physiologic recovery—functional capacity, toxicity clearance, and nutritional status. Second, perform integrated oncologic reassessment with contrast-enhanced CT and CA19-9 kinetics as key decision-support elements; normalization or robust decline is reassuring, while persistent elevation signals unfavorable biology. Third, consider staging laparoscopy to exclude occult peritoneal disease. Importantly, exploration should not be abandoned solely due to persistent vascular contact, stable imaging, or modest regression, provided there is no metastatic progression and the holistic clinical-biological assessment remains favorable. For chemotherapy-based protocols, surgical exploration is typically performed 4–8 weeks after the last cycle; radiotherapy-containing regimens may favor earlier intervention (as noted in Section 4.2).

## 6 Staging laparoscopy and beyond: optimizing patient selection

Staging laparoscopy is explicitly endorsed by NCCN guidelines to exclude occult peritoneal, hepatic, or extra-regional nodal disease before proceeding to resection (8). In a Mayo Clinic series of 1,004 localized PDAC patients, staging laparoscopy detected occult peritoneal disease in 18% overall, with a post-NAT yield of 14%—clinically substantial despite being lower than the 22% observed upfront (54). A multicenter BRPC cohort similarly identified occult metastases in 10% of cases, steering patients away from non-therapeutic laparotomy (55). Its post-NAT value lies in detecting interval metastatic progression, enabling timely avoidance of futile exploration (56). Emerging molecular techniques—such as KRAS ctDNA detection in peritoneal lavage—and optical adjuncts like ICG fluorescence remain investigational, lacking standardization and PDAC-specific validation (57, 58). Thus, staging laparoscopy continues to offer meaningful yield after NAT, with its principal value being the prevention of non-therapeutic resection and the reinforcement of biological selection prior to major pancreatic surgery.

## 7 Vascular management after NAT: venous, arterial, and artery-sparing strategies

**Figure 2:**
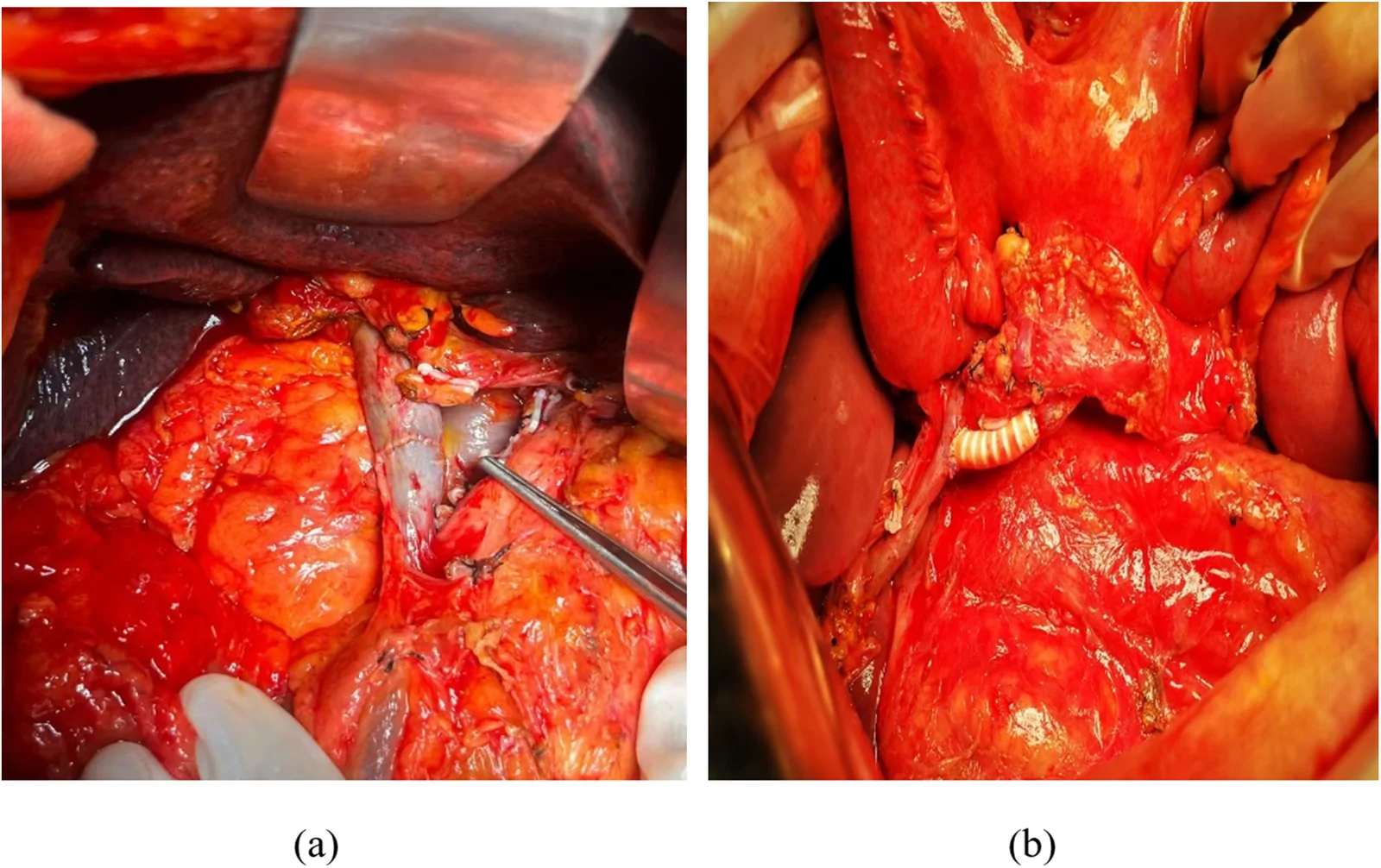
**(a)** Intraoperative views of pancreaticoduodenectomy (PD) with combined reconstruction of the superior mesenteric vein (SMV); **(b)** SMV end-to-end anastomosis after segmental resection.

For patients proceeding to resection after NAT, securing an R0 margin (tumor-free margin >1 mm) remains the cornerstone of improved survival and reduced local recurrence (59). In BRPC, this goal frequently necessitates *en bloc* resection of involved vascular structures—most commonly portal-mesenteric venous resection, with arterial resection reserved for highly selected cases. Figure 1 illustrates representative examples from our institutional experience, depicting combined SMV and CHA resections with end-to-end reconstruction (Figures 2, a,b), underscoring the importance of centralized, multidisciplinary care in managing these complex procedures.

### 7.1 Venous resection: techniques, outcomes, and selective use

Venous resection (VR) is required in 28.6% of BRPC patients undergoing resection—nearly seven times the rate in anatomically resectable disease (4.0%)—underscoring how venous involvement drives both classification and surgical complexity (60). The International Study Group of Pancreatic Surgery (ISGPS) has codified VR indications with a four-grade classification (venorrhaphy, patch venoplasty, segmental resection with end-to-end anastomosis, and interposition grafting), establishing VR as a routine intraoperative tool (61). The choice of technique depends on the extent and geometry of venous encasement, with interposition grafting reserved for cases where tension-free end-to-end anastomosis is not feasible (60, 61). Benchmarking data from a large European series (*n* = 2,265; VR in 694) demonstrate that PD with VR is safe in high-volume centers (mortality <4%, major morbidity up to 36%), though interposition grafting carries the highest rates of major morbidity and portal vein thrombosis; median OS in margin-negative cases was 23 months (61). After propensity adjustment, neither VR technique nor reconstruction method independently predicted morbidity or survival; tumor biology, patient characteristics, and R0 status remain the principal determinants of outcome, suggesting that VR's prognostic impact is a surrogate for aggressive tumor biology rather than a direct consequence of the vascular intervention itself (60).

In the post-NAT setting, venous preservation confers comparable survival to VR with superior patency, arguing against routine VR when R0 appears achievable without it (62). In summary, VR remains indispensable for achieving R0 in selected BRPC patients after NAT, but its application should be selective—balancing anatomic necessity against reconstruction-related morbidity—rather than routine.

### 7.2 Venous shunts and bypasses: indications and outcomes

Long-segment involvement of the portal-mesenteric venous axis, particularly with extension into jejunal/ileal tributaries or cavernous transformation, poses exceptional challenges for reconstruction. Recent technical descriptions outline three parallel strategies: temporary bypasses, permanent interposition grafts, and elective shunts between portal and systemic or splenic and renal circulations (63–66). A 2024 systematic review (145 patients) reported an overall morbidity of 30% and long-term patency of 83% for definitive shunts (67). In a recent high-volume series, temporary alloplastic venous bypass (63 cases) achieved comparable early outcomes between mesoportal and mesocaval approaches, with selection individualized to anatomy and anticipated arterial work (68, 69). When graft-based reconstruction is not feasible, permanent mesocaval shunt has been used as a salvage procedure with acceptable early safety (70, 71); in select cases with robust collaterals, VR without reconstruction has also been described (72). Temporary bypass is most useful when prolonged clamping or complex branch involvement threatens mesenteric drainage, whereas permanent shunting should be reserved for salvage situations where standard reconstruction is not feasible or venous decompression is required.

### 7.3 Arterial resection: cautious use and evolving outcomes

Major guidelines remain cautious regarding arterial resection (AR). ESMO (2023) generally does not recommend AR after induction therapy but allows case-by-case consideration in experienced centers (11), while NCCN (v2.2025) restricts AR to very select patients (8). The 2024 REDISCOVER consensus advocates AR for celiac or hepatic artery involvement but advises against routine SMA resection (73). Thus, AR is warranted only when a distinct oncological gain is expected, after multidisciplinary evaluation, and in high-volume institutions with vascular surgical expertise (8, 11, 73). In practice, AR is rare: a Dutch national cohort reported 1.4% (54/3,868) (74), and a major European center reported 1.0% (39/3,953) over two decades (75). Historically associated with high 90-day complication and mortality rates—mainly from hemorrhage, pancreatic leakage, and mesenteric ischemia (74, 76)—a recent meta-analysis (2018-2024) found perioperative mortality roughly three times higher with AR than with artery-sparing procedures, though outcomes have improved with centralization and growing expertise (77). Institutional data confirm a learning curve: Heidelberg's 385-case program saw in-hospital mortality drop from 8.8% to 4.8% post-2013, with a per-surgeon learning curve of 15 ARs (78); another 236-procedure series documented 30- and 90-day mortality of 7.2% and 9.7%, with a program-level inflection after 106 ARs and better long-term results in the recent NAT era (79).

### 7.4 Periarterial divestment: an emerging artery-sparing option

Periarterial divestment (PAD) and related sub-adventitial maneuvers—exploiting the plane between the adventitia and the external elastic lamina—have gained attention as alternatives to formal arterial resection, particularly when NAT-induced fibrosis creates a favorable dissection plane (80, 81). Available early experience suggests that PAD is associated with lower rates of POPF, post-pancreatectomy hemorrhage, ischemic complications, and reoperation compared with formal arterial resection, though higher reported R0 rates may reflect favorable tumor biology and patient selection rather than inherent technical superiority (78, 82). A comparative study of celiac axis involvement found that distal pancreatectomy with celiac divestment achieved comparable R0 rates, recurrence patterns, and OS to *en bloc* arterial resection, but with reduced POPF, intra-abdominal infection, and hepatic ischemia (83). In a cohort of 125 BRPC patients, PAD achieved 90-day mortality of 3.2%, median OS of 20.6 months, and 1-/3-year survival of 73.2%/22.9%; intraoperative Neuro-Patch reinforcement was associated with lower post-pancreatectomy hemorrhage (2% vs. 13.5%) (84).

Despite these encouraging results, the evidence remains limited to small, single-center, retrospective series with highly selected patients. No prospective comparative data exist to establish oncologic equivalence to arterial resection. PAD is technically demanding, requiring high-volume center expertise and contingency planning for conversion to formal arterial resection if full-thickness invasion is encountered. Until larger prospective studies are available, divestment should be reserved for patients with a distinct periarterial or sub-adventitial plane suggesting no transmural invasion; if full-thickness invasion is identified intraoperatively, the procedure should be abandoned and formal arterial resection reconsidered based on operative risk, disease biology, and R0 likelihood.

## 8 Intraoperative frozen section: contested evidence, site-specific utility, and calibrated use

Intraoperative frozen section (FS) serves to assess margins at key transection sites—particularly the pancreatic neck and perivascular surfaces—to guide additional resection in pursuit of R0 (8). Its oncologic benefit remains debated, with some studies reporting improved local control and others no survival advantage (85–87). NAT adds complexity through treatment-induced fibrosis and inflammation, though recent data indicate that diagnostic accuracy is not diminished after preoperative therapy (86, 88, 89). The utility of FS varies by sampling site: at the pancreatic neck, a multicenter cohort of 272 NAT-treated patients found that converting a positive FS to negative did not improve survival, suggesting a positive result reflects adverse tumor biology rather than a correctable technical issue (90). Perivascular FS assessment is particularly challenging; an 85-case series reported 38% sensitivity, 100% specificity, and a 62% false-negative rate, meaning a negative result does not reliably exclude vessel involvement (91). A positive perivascular FS, however, is regarded as conclusive and, per REDISCOVER guidelines, should prompt either *en bloc* vascular resection or deferral of radical resection (92, 93).

In the NAT era, FS should serve as a focused decision-aid, not justification for exhaustive margin revision. A positive perivascular FS is clinically meaningful and guides a binary decision—vascular resection or deferral—whereas a negative result offers limited reassurance due to sampling constraints. Critically, in NAT-treated patients, a positive margin often reflects underlying tumor biology rather than a purely technical deficit, a distinction that should inform both intraoperative strategy and overall therapeutic planning.

## 9 Perioperative safety of NAT: procedure-specific benefits and risks

A consistent finding across meta-analyses and large cohorts is that NAT does not adversely affect early postoperative safety after pancreaticoduodenectomy (PD). In high-risk PD, NAT has been linked to a measurable reduction in clinically relevant CR-POPF, possibly due to tumor downsizing, reduced inflammation, and fibrotic stiffening of the gland (94, 95). This protective profile was confirmed in an international collaboration pooling 11,402 PDs, where NAT correlated with fewer CR-POPFs, interventions, and infectious complications—benefits that persisted with vascular resection (96). However, the impact of NAT on POPF risk depends on both modality and resection type. The protective signal is stronger with chemoradiation than chemotherapy alone (97, 98), but does not extend to distal pancreatectomy (DP): meta-analysis and an international propensity-matched cohort both found no reduction in POPF, severe complications, reoperation, or 90-day mortality after DP (99, 100). A notable caveat is an increased risk of postoperative deep-vein thrombosis (DVT) after DP in NAT-treated patients, emphasizing the need for vigilant thromboprophylaxis in this subgroup (101).

In summary, NAT does not increase short-term morbidity or mortality after pancreatectomy. For PD, it confers a protective advantage, particularly in reducing CR-POPF; for DP, no such benefit is observed, though the overall perioperative profile remains comparable to upfront surgery except for elevated DVT risk. These findings support proceeding with NAT without undue concern for elevated perioperative risk, while maintaining procedure-specific prevention strategies.

## 10 Antithrombotic management: guidelines, evidence gaps, and individualized practice

Antithrombotic management after pancreatectomy—particularly with venous reconstruction and NAT—requires stratification into standard venous thromboembolism (VTE) prophylaxis and reconstruction-driven protocols. Current guidelines (ITAC, ESMO, ASCO) uniformly advise pharmacological prophylaxis for hospitalized cancer patients and extended prophylaxis up to 4 weeks after major abdominopelvic surgery, but pancreas-specific evidence is lacking (102–104). The ERAS and AHPBA guidelines acknowledge this gap, noting that early bleeding risk may outweigh thrombotic events postoperatively (105, 106). This uncertainty is reflected in wide practice variation across centers (107). A systematic review of venous reconstruction (23 studies, *n* = 2,751) found thrombosis risk lowest after venorrhaphy/end-to-end anastomosis and highest with interposition grafts, though heterogeneity in anticoagulation regimens precluded pooled estimates (108). For venous stenting, pooled analysis showed no patency benefit from antithrombotic therapy (109), while registry data (*n* = 972) suggested early therapeutic anticoagulation lowered 30-day PV thrombosis but had no sustained effect at 90 days (110). For arterial reconstruction, evidence is even thinner, with no validated pancreas-tailored algorithms (111).

Based on available evidence, we propose the following pragmatic framework:

1. Standard VTE Prophylaxis: Pharmacological prophylaxis (e.g., LMWH) starting 12–24 h postoperatively, provided no active bleeding.
2. Reconstruction-Type Stratification:
  - **Low thrombotic risk** (venorrhaphy, patch angioplasty, end-to-end anastomosis): Prophylactic-dose anticoagulation is sufficient.
  - **High thrombotic risk** (interposition grafts, extensive/arterial reconstruction): Consider therapeutic-dose anticoagulation 24–48 h postoperatively, guided by bleeding status and stable hemoglobin, individualized with multidisciplinary input.
  - **Timing and Duration**: Continue prophylaxis until discharge; for high-risk reconstructions, consider extended prophylaxis (up to 4 weeks) or therapeutic anticoagulation for 3–6 months, though evidence is limited.

In summary, routine full anticoagulation is not substantiated. Clinical choices should be made case-by-case, weighing reconstruction technique, hemorrhagic risk, and stable hemostasis.

## 11 Conclusions

NAT has redefined BRPC as a disease pathway gated by tumor biology and sequenced according to therapeutic response. Converging evidence now establishes NAT as the preferred initial strategy, improving ITT survival while selecting against early systemic progression. For the practicing surgeon, this paradigm shift demands disciplined, standardized post-NAT decision-making. Operability should be assessed through a sequential physiologic, oncologic, and surgical filter—with contrast-enhanced CT and CA19-9 kinetics as the pragmatic foundation, and staging laparoscopy to exclude occult peritoneal disease before high-stakes exploration. Exploration should proceed with a selective, margin-based approach to vascular management: portomesenteric venous resection with mandatory reconstruction is established; arterial resection is reserved for rigorously selected patients at experienced centers; periarterial dissection is a vessel-sparing option when the plane is favorable, with strict criteria for aborting if transmural invasion is encountered. The proposed algorithm (Figure 1) encapsulates these decision points. Future progress will require stratified prospective studies with harmonized definitions, prespecified restaging criteria, and robust evaluation of reconstruction-specific anticoagulation protocols.
